# Supplementary material for: Analysis of PPARγ Signaling Activity in Psoriasis
Source: Int J Mol Sci. 2021 Aug 10;22(16):8603. doi: 10.3390/ijms22168603 (PMC8395241; doi:10.3390/ijms22168603)
Supplement: Supplementary file 1 [file ijms-22-08603-s001.zip › Supplemental materials_Analysis of PPARg signaling activity in psoriasis/Pathway models/Models images and html files/PPARG expressed in cells assotiated with psoriasis/225584.html]

lymphocyte


# CellType lymphocyte

|  |  |
| --- | --- |
| URN | urn:agi-ncimcelltype:C1550651 |
| Total Entities | 81 |
| Connectivity | 9905 |
| Name | lymphocyte |
| ObjectType | Cell |

---

|  |  |
| --- | --- |
| ChildConcepts | lymphocytotoxicity |
|  | lymphocyte function |
|  | lymphocyte adhesion |
|  | cell mediated cytotoxicity |
|  | lymphocyte structure |
|  | lymphocyte aggregation |
|  | lymphocyte migration |
|  | lymphocytic blood cell differentiation |
|  | lymphocyte homeostasis |
|  | lymphocyte proliferation |
|  | lymphocyte apoptosis |
|  | lymphocyte chemotaxis |
|  | lymphocyte anergy |
|  | lymphocyte proliferative response |
|  | lymphocyte homing |
|  | T-cell |
|  | B-cell |
|  | natural helper cell |
|  | mesenteric lymph node lymphocyte |
|  | spleen lymphocyte |
|  | lymphomonocyte |
|  | killer cell |
|  | null cell |
|  | tumor-infiltrating lymphocyte |
|  | large granular lymphocyte |
|  | peripheral lymphocyte |
|  | cytotoxic lymphocyte |
|  | lymphocytic infiltration |
|  | lymphocyte population |
|  | lymphocyte survival |
|  | lymphocyte division |
|  | lymphocyte phenotype |
|  | lymphocyte rolling |
|  | lymphocyte motion |
|  | lymphocyte development |
|  | lymphocyte extravasation |
|  | lymphocyte sequestration |
|  | lymphocyte transendothelial migration |
|  | lymphocyte diapedesis |
|  | lymphocyte formation |
|  | lymphocyte fate |
|  | lymphocyte priming |
|  | lymphocyte selection |
|  | lymphocyte invasion |
|  | lymphocyte motility |
|  | lymphocyte tethering |
|  | lymphocyte compartmentalization |
|  | lymphocyte localization |
|  | lymphocyte recognition |
|  | lymphocyte polarity |
|  | lymphocyte renewal |
|  | lymphocyte growth |
|  | lymphocyte distribution |
|  | lymphocyte fate commitment |
|  | lymphocyte death |
|  | lymphocyte interaction |
|  | lymphocyte volume |
|  | lymphocyte T-cell homeostasis |
|  | lymphocyte repertoire |
|  | lymphocyte phagocytosis |
|  | lymphocyte disruption |
|  | lymphocyte morphogenesis |
|  | lymphocyte communication |
|  | lymphocyte contact |
|  | lymphocyte ratio |
|  | lymphocyte spreading |
|  | lymphocyte lifespan |
|  | lymphocyte stress |
|  | lymphocyte phagocyte activity |
|  | lymphocyte damage |
|  | lymphocyte destruction |
|  | lymphocyte macrophage differentiation |
|  | lymphocyte size |
|  | lymphocyte senescence |
|  | lymphocyte autophagy |
|  | lymphocyte transdifferentiation |
|  | lymphocyte swelling |
|  | lymphocyte ultrastructure |
|  | lymphocyte transepithelial migration |
|  | lymphocyte regeneration |
|  | lymphocyte anchorage |

---

|  |  |
| --- | --- |
| Pathway | Proteins/Ions Transporters -> Expression Targets in Lymphoid System and Blood |
|  | LDLR -> Expression Targets in Lymphoid System and Blood |
|  | TGF-beta Receptors Family -> Expression Targets in Lymphoid System and Blood |
|  | EGFR-> Expression Targets in Lymphoid System and Blood |
|  | Elevated Receptors -> Expression Targets in Lymphoid System and Blood |
|  | CD Markers -> Expression Targets in Lymphoid System and Blood |
|  | VIPR1/VIPR2 -> Expression Targets in Lymphoid System and Blood |
|  | TNF Receptors -> Expression Targets in Lymphoid System and Blood |
|  | Immunoglobulin Superfamily -> Expression Targets in Lymphoid System and Blood |
|  | Receptor Tyrosine Kinase Family -> Expression Targets in Lymphoid System and Blood |
|  | C5AR1 -> Expression Targets in Lymphoid System and Blood |
|  | IgE Receptors -> Targets in Lymphoid System and Blood |
|  | Chemokine Receptor Family -> Expression Targets in Lymphoid System |
|  | PPARG expressed in cells assotiated with psoriasis |
|  | Neighbors of lymphocyte |

---

|  |  |
| --- | --- |
| MedScan ID | 10000091 |

---

|  |  |
| --- | --- |
| Alias | lymphocyte |
|  | lymphocytes |
|  | lymphocytic |
|  | lymphocytal |

---

|  |  |
| --- | --- |
| NCIm ID | C1550651 |

---
